# Variations by ethnicity in referral and treatment pathways for IAPT service users in South London

**DOI:** 10.1017/S0033291721002518

**Published:** 2023-02

**Authors:** Hannah Harwood, Rebecca Rhead, Zoe Chui, Ioannis Bakolis, Luke Connor, Billy Gazard, Jheanell Hall, Shirlee MacCrimmon, Katharine A. Rimes, Charlotte Woodhead, Stephani L. Hatch

**Affiliations:** 1 Department of Psychological Medicine, Institute of Psychiatry, Psychology & Neuroscience, King's College London, London, UK; 2 Department of Biostatistics & Health Informatics, Institute of Psychiatry, Psychology & Neuroscience, King's College London, London, UK; 3 Health Service & Population Research Department, Centre for Implementation Science, Institute of Psychiatry, Psychology & Neuroscience, King's College London, London, UK; 4 Department of Psychology, Institute of Psychiatry, Psychology & Neuroscience, King's College London, London, UK; 5 Economic and Social Research Council (ESRC) Centre for Society and Mental Health, King's College London, London, UK

**Keywords:** Ethnic groups, IAPT, inequalities, mental health, therapy

## Abstract

**Background:**

The Improving Access to Psychological Therapies (IAPT) programme aims to provide equitable access to therapy for common mental disorders. In the UK, inequalities by ethnicity exist in accessing and receiving mental health treatment. However, limited research examines IAPT pathways to understand whether and at which points such inequalities may arise.

**Methods:**

This study examined variation by ethnicity in (i) source of referral to IAPT services, (ii) receipt of assessment session, (iii) receipt of at least one treatment session. Routine data were collected on service user characteristics, referral source, assessment and treatment receipt from 85 800 individuals referred to South London and Maudsley NHS Foundation Trust IAPT services between 1st January 2013 and 31st December 2016. Multinomial and logistic regression analysis was used to assess associations between ethnicity and referral source, assessment and treatment receipt. Missing ethnicity data (18.5%) were imputed using census data and reported alongside a complete case analysis.

**Results:**

Compared to the White British group, Black African, Asian and Mixed ethnic groups were less likely to self-refer to IAPT services. Black Caribbean, Black Other and White Other groups are more likely to be referred through community services. Almost all racial and minority ethnic groups were less likely to receive an assessment compared to the White British group, and of those who were assessed, all racial and ethnic minority groups were less likely to be treated.

**Conclusions:**

Racial and ethnic minority service users appear to experience barriers to IAPT care at different pathway stages. Services should address potential cultural, practical and structural barriers.

## Introduction

Common mental disorders (CMDs) such as depression and anxiety cause considerable burden to both individuals and the economy, with an estimated 72 million working days lost each year (Centre for Mental Health, 2017). In England alone, one in six adults experience a CMD in a given week (Mcmanus, Bebbington, Jenkins, & Brugha, 2016). Left untreated, CMDs can result in poor physical, social and occupational functioning and premature death (Stansfeld, Fuhrer, & Head, 2011; Zivin et al., 2015). In the UK, there are ethnic inequalities in seeking and receiving mental health treatment (Cooper et al., 2013; Grey, Sewell, Shapiro, & Ashraf, 2013; Sizmur & McCulloch, 2016). Racial and ethnic minority groups may have an increased vulnerability to CMDs through experiences of racism and discrimination (Hatch et al., 2016; Karlsen, Nazroo, McKenzie, Bhui, & Weich, 2005; Wallace, Nazroo, & Bécares, 2016), and being more likely to experience social inequalities that can contribute to mental ill-health (Allen, Balfour, Bell, & Marmot, 2014; Marmot & Bell, 2012). Delayed access to psychological support for CMDs can have a substantial negative impact on quality of life and functioning and can lead to CMDs developing into disorders more difficult to treat (Stansfeld et al., 2011).

In 2007, only a quarter of individuals diagnosed with CMDs were receiving appropriate specialist care in the UK (Layard, Clark, Knapp, & Mayraz, 2007). As a result, the Improving Access to Psychological Therapies (IAPT) programme was launched to provide equitable access to evidence-based psychological interventions for people experiencing CMDs (Clark, 2011). Each clinical commissioning group across England is responsible for funding health services for their local area and providing their own IAPT services (NHS Digital, 2020). As such, there may be slight area level variations in the way these services are run, i.e. location of service delivery (GP surgeries, hospitals, community centres), the interventions available and level of service advertisement (British Association for Counselling and Psychotherapy, 2016; National Collaborating Centre for Mental Health, 2020) – all of which potentially impact on performance indicators such as waiting times and treatment outcomes. It is therefore important to also consider how the area in which service users live, i.e. their borough or locality of IAPT service, may impact treatment pathways.

Despite evidence of ethnic inequalities in wider mental health service use, there is a lack of research into referral and treatment pathways for racial and ethnic minority service user groups accessing IAPT. Of the limited existing research, studies examining ethnic differences in IAPT access and outcomes used data from the initial IAPT pilot sites between 2006 and 2010, which may now present an outdated representation of IAPT services. These initial studies found racial and ethnic minority groups were underrepresented in IAPT services, being less likely to be referred into the service than White groups (de Lusignan, Chan, Parry, Dent-Brown, & Kendrick, 2012; Parry et al., 2011.), and greater proportions of Black and Asian service users accessed services via self-referral rather than their general practitioner (GP) (Clark et al., 2009; Parry et al., 2011.). An IAPT service in south London offering a self-referral option between 2009 and 2010 was shown to lead to more equitable access to psychological therapies for racial and ethnic minority groups compared to GP-referral (Brown et al., 2014). This reflects inequalities in wider mental health service use among racial and ethnic minority groups in the UK; survey findings also suggest that racial and ethnic minority groups are less likely to seek help for CMDs through primary care than their White counterparts (National Psychiatric Morbidity Survey – Cooper et al., 2013), or to receive any treatment for mental ill-health (medication, counselling or both), with Black groups least likely to receive treatment (Adult Psychiatric Morbidity Survey Mcmanus et al., 2016). All IAPT service users are now able to self-refer. Analysis of current IAPT data is required to determine whether and the extent to which such inequalities in service provision still persist.

Identifying inequalities in referral and treatment pathways of IAPT services for racial and ethnic minority service users is crucial for ensuring equity of access and the provision of appropriate, evidence-based NHS mental health care for these groups. The current study aimed to examine variation by ethnicity in (1) source of referral, (2) receipt of an initial assessment following referral, and (3) receipt of at least one treatment session within an IAPT service. The impact of the area (specific borough) of IAPT service on outcomes was examined for each aim. We hypothesised that compared to the White British service user group, IAPT service users from racial and ethnic minority groups would be (i) more likely to self-refer than be referred by a GP, (ii) less likely to receive an assessment and (iii) less likely to receive a treatment session.

## Method

### Setting and data source

The South London and Maudsley (SLaM) NHS Foundation Trust provides access to psychological therapies across four South London boroughs; Croydon, Lambeth, Lewisham and Southwark. Each borough implements their own IAPT services, with the majority of referrals coming from GPs or via self-referral. The service implements a stepped-care model to ensure that service users are offered the least-intrusive appropriate intervention first (National Collaborating Centre for Mental Health, 2020). Low intensity interventions may include self-help programmes, online cognitive behavioural therapy or group interventions. High intensity treatments are often a form of individual therapy but can include other intervention methods. Service users can be stepped up to high intensity or stepped down to low intensity as required (NHS Digital, 2018a).

IAPT services provide treatment for people with common mental health problems, including; depression, generalised anxiety disorder, social anxiety disorder, panic disorder, agoraphobia, OCD, phobias, PTSD, health anxiety and body dysmorphic disorder. For more information on referral criteria please see online Supplementary Material B.

Routine clinical data from the IAPTus electronic service user database (http://www.iaptus.co.uk) were exported to the Clinical Record Interactive Search (CRIS) system at SLaM, which provides pseudo-anonymised electronic health record data for the purposes of research analysis (Stewart et al., 2009).

### Participants

Participants were adults (aged 16 years and older) who had been referred into IAPT services provided by SLaM between 1st January 2013 (when all four boroughs had established IAPT services) and 31st December 2016 (*N* = 85 800). Some individuals were referred more than once during the specified time period. To ensure independence of data, only the first treatment episode per person was included in the analysis.

### Measure of referral source

Referral source was extracted from structured fields in IAPTus and were categorised into GP referral, self-referral, secondary health services or community service referral. Secondary health services included secondary mental health services, hospital services and outpatient clinics. Community services included statutory services such as Job Centre Plus (a government-funded employment agency), voluntary organisations, education providers and criminal justice (prison and probation services).

### Measure of assessment and treatment

If there was at least one service user session record that had a purpose of ‘assessment’ and it was attended, the service user was categorised as having received an assessment (1 = assessed, 0 = not assessed). A service user was categorised as having received treatment if at least one of their session records had a purpose of ‘treatment’ (1 = treated, 0 = not treated). The latter analysis was restricted to those who had been assessed.

### Measures of demographic characteristics

Service user gender (male, female) and exact age were recorded in IAPTus. Age was collapsed into age bands for descriptive purposes (16–24, 25–34, 35–44, 45–54, 55–64 and 65+ years), exact age was used for all age-adjusted models. Information on the ethnicity of the service user was collected at triage or initial assessment. The 17 ethnicity categories from the UK Census and used in IAPTus were recoded into White British, Black Caribbean, Black African, Black Other, Asian, Mixed, White Other and Other. The black ethnic group was disaggregated into three categories because sample size was sufficient enough to do so and we felt it important to explore black ethnicities separately due to their distinct experiences.

### Measures of mental health

#### Patient health questionnaire depression scale

Symptoms of depression were measured using the validated nine-item Patient Health Questionnaire (PHQ-9; Kroenke, Spitzer, and Williams, 2001). A PHQ-9 score ⩾10 is considered to be of clinical significance (sensitivity of 88% and a specificity of 88% for major depression) and is used as a cut-off to identify caseness in IAPT (National Collaborating Centre for Mental Health, 2020). Both the internal consistency and test−retest reliability of the PHQ-9 is excellent (Cronbach *α* = 0.89, intraclass correlation = 0.84).

#### Generalised anxiety disorder scale

Symptoms of anxiety were measured using the validated seven-item generalised anxiety disorder assessment (GAD-7; Spitzer, Kroenke, Williams, and Löwe, 2006). A score of ⩾8 has a sensitivity of 89% and a specificity of 82% and is used as a cut-off point for caseness in IAPT (National Collaborating Centre for Mental Health, 2020). The internal consistency of the GAD-7 is excellent (Cronbach *α* = 0.92). Test−retest reliability is also good (intraclass correlation = 0.83).

For the purposes of these analyses, scores collected at the initial contact stage, prior to assessment, were used to measure baseline symptoms of depression and anxiety.

### Statistical analysis

#### Missing data

A total of 85 800 SLaM IAPT service users from 2013 to 2016 over the age of 16 were identified. There was missing data on some outcome and exposure variables – 0.7% of the sample (*n* = 593) had missing data on method of referral, 0.1% (*n* = 91) of the sample had missing data on gender and 18.5% (*n* = 15 917) had missing ethnicity data.

Though the amount of missing data for method of referral and gender is negligible, the amount of missing ethnicity data is substantial, particularly as ethnicity is the focal point of this study. Low levels of recording for ethnicity is an issue that typically constrains studies using health record data (Aspinall & Jacobson, 2007; Kumarapeli, Stepaniuk, De Lusignan, Williams, & Rowlands, 2006; Mathur et al., 2014). Often, missing ethnicity data are addressed by removing ethnicity from the analysis entirely (complete case analysis) (Osborn et al., 2015), or by performing single imputation of missing values with the White ethnic group (Hippisley-Cox et al., 2008) − these methods generally lead to biased estimates of association and standard errors (Sterne et al., 2009). Multiple imputation (MI) is another common method of addressing missing data. However, the probability that ethnicity is recorded in primary care may well vary systematically by ethnic group, even after adjusting for other variables (Mathur et al., 2014).This implies a potential missing not at random (MNAR) mechanism for ethnicity, and as a result, standard MI might fail to give valid reference for the underlying population.

Weighted MI can be used to address the specific problem of MNAR ethnicity data in health records and overcome the limitations of the more commonly used methods mentioned (Pham, Morris, & Petersen, 2015). Weighted MI combines MI and probability weights which are calculated using marginal population distribution of ethnicity available in the UK census data. Census summary statistics for ethnicity provide weights which inform the MI such that the imputed dataset better reflects the ethnicity of the population in question (in this instance, residents of the four boroughs which comprise SLaM) and not that of the complete data. Several studies have found this to reduce bias compared to standard MI methods (Pham et al., 2015; Pham, Carpenter, Morris, Wood, & Petersen, 2019). This method assumes that a particular service user group is somewhat representative of the population, which may not always be the case. Racial and ethnic minority populations experience barriers to care and are less likely to engage with health services (Cooper et al., 2013; Mcmanus et al., 2016). However, self-referral options (such as those provided by IAPT) have been shown to lead to more equitable provision of psychological therapies for racial and ethnic minority groups compared to GP-referral (Brown et al., 2014; Parry et al., 2011.). Therefore, although this weighted MI approach may overestimate proportions of racial and ethnic minority service users (because these populations can be underrepresented in healthcare), IAPT's self-referral options may mitigate against this underrepresentation.

To ensure a robust analysis, this study reports the findings of analyses from (i) complete case data and (ii) an imputed dataset where ethnicity has been imputed using weighted MI, utilising 2011 census data on ethnicity for Croydon, Lambeth, Lewisham and Southwark (see online Supplementary Material C for these Census data). In addition, analysis of data where ethnicity has been imputed using a more standard approach – multiple imputation with chained equations (MICE) (White, Royston, & Wood, 2011) – will be reported in online Supplementary Material D. Findings from the MICE imputed dataset will be commented on in manuscript if they contradict findings from either complete case or weighted mi datasets.

The proportion of missing ethnicity data varied across boroughs; 6% of data from Lewisham was missing, 8% from Lambeth, 30% from Croydon and 34% from Southwark. As such, the socio-demographic characteristics and prevalence of anxiety and depression among service users, as well as the main outcomes of this study, will be broken down by borough.

#### Analysis

Data analyses were conducted using Stata 15 (StataCorp, 2019). Descriptive statistics were calculated to describe the analytic sample by ethnicity, age, gender, borough of service, depression and anxiety symptoms, referral source, receipt of an assessment and of at least one treatment session (among those assessed). Due to each borough implementing its own IAPT service and the potential differences this may pose for service user pathways to treatment, borough was adjusted for separately to explore the impact of borough of service on the outcomes of interest. Therefore, to examine variation by ethnicity in IAPT referral source, multinomial regression analyses were conducted; unadjusted (model 1), adjusting for age and gender and year of referral (model 2), and adjusting to additionally include borough of service (model 3). Relative risk ratios (RRRs) with 95% confidence intervals (CI) are reported. Next, logistic regression analysis was used to determine whether ethnicity was associated with (i) receiving an assessment session, and (ii) receipt of at least one IAPT treatment session (among those who were assessed). Odds ratios (ORs) with 95% CI are reported. These analyses were also adjusted for age, gender, year of referral and borough in the same manner. Interaction effects were also tested using a Wald test to compare models with and without an interaction term to determine whether borough moderated the effect of ethnicity on any of the main outcomes.

In our examination of variations in referral source, assessment, and treatment by ethnicity, we will first report findings where there were no discrepancies between imputed and complete case data and then highlight discrepant findings.

## Results

The characteristics of the sample are shown in Table 1. The majority of the sample identified as White British (52.4%), female (63.0%) and were referred to IAPT services in Lambeth (30.9%). The largest racial and ethnic minority groups were Black Caribbean (12.0%) and White Other (12.4%), and over a third of the sample were aged between 25 and 34 years (33.5%). Upon referral, 77.6% of the sample met caseness for depression and 82.2% for anxiety. Most referrals to IAPT services were via primary care (54.3%) or self-referral (40.3%). The majority of the sample had received an assessment session (64.9%) and of those, over two-thirds received at least one treatment session thereafter (70.4%). See online Supplementary Material A for a breakdown of sample characteristics by ethnicity.

**Table 1.** Characteristics of service users aged 16 + referred to IAPT services between 2013 and 2016 across the four London borough that comprise SLaM

	Overall sample (complete case)		Croydon		Lambeth		Lewisham		Southwark	
	*N*	%	*N*	%	*N*	%	*N*	%	*N*	%
Ethnicity										
White British	36 640	52.4	5940	54.3	12 712	52.1	10 179	50.2	7809	54.6
Black Caribbean	8381	12.0	1152	10.5	3337	13.7	2528	12.5	1364	9.5
Black African	3508	5.0	503	4.6	817	3.4	1427	7.0	761	5.3
Black Other	1601	2.3	187	1.7	671	2.8	338	1.7	405	2.8
Asian	5153	7.4	1236	11.3	1708	7.0	1206	5.9	1003	7.0
White Other	8664	12.4	900	8.2	2997	12.3	2967	14.6	1800	12.6
Mixed	4387	6.3	710	6.5	1584	6.5	1250	6.2	843	5.9
Other	1549	2.2	309	2.8	552	2.3	374	1.8	314	2.2
Age										
16–24	15 099	17.6	2633	16.8	4322	16.3	4185	19.2	3959	18.2
25–34	28 738	33.5	4329	27.5	10 397	39.2	6634	30.5	7378	33.8
35–44	18 117	21.1	3355	21.3	5530	20.9	4586	21.1	4646	21.3
45–54	13 783	16.1	2990	19.0	3908	14.7	3484	16.0	3401	15.6
55–64	6430	7.5	1479	9.4	1630	6.1	1733	8.0	1588	7.3
65+	3633	4.2	933	5.9	726	2.7	1138	5.2	836	3.8
Gender										
Male	31 611	36.9	5528	35.2	10 206	38.5	7924	36.5	7953	36.5
Female	54 098	63.1	10 157	64.8	16 302	61.5	13 814	63.5	13 825	63.5
Borough										
Croydon	15 719	18.3	–	–	–	–	–	–	–	–
Lambeth	26 513	30.9	–	–	–	–	–	–	–	–
Lewisham	21 760	25.4	–	–	–	–	–	–	–	–
Southwark	21 808	25.4	–	–	–	–	–	–	–	–
PHQ9 caseness										
Non-caseness	14 082	22.4	2023	22.8	3951	21.9	4115	19.2	3993	27.7
Caseness	48 689	77.6	6837	77.2	14 096	78.1	17 359	80.8	10 397	72.3
GAD-7 caseness										
Non-caseness	11 136	17.8	1554	17.6	2758	15.3	3476	16.2	3348	23.3
Caseness	51 594	82.2	7294	82.4	15 286	84.7	17 983	83.8	11 031	76.7
Referral source^a^										
Primary care	46 290	54.3	9462	60.5	6834	25.9	13 019	60.2	16 975	78.8
Self-referral	34 368	40.3	5377	34.4	17 080	64.7	7925	36.7	3986	18.5
Secondary care	2759	3.2	784	5.0	1016	3.9	386	1.8	573	2.7
Community services	1790	2.1	29	0.2	1455	5.5	286	1.3	20	0.1
Assessment received										
No	30 230	35.2	6878	43.8	8429	31.8	7511	34.5	7412	34.0
Yes	55 570	64.8	8841	56.2	18 084	68.2	14 249	65.5	14 396	66.0
Treatment received										
No	16 445	29.6	3500	39.6	5482	30.3	2305	16.2	5158	35.8
Yes	39 125	70.4	5341	60.4	12 602	69.7	11 944	83.8	9238	64.2

Community services include: voluntary sector organisations, government service providers, education providers and criminal justice referrals (prison and probation services).

a Missing referral method data on *n* = 462.

### Variations in referral source

#### Self-referral

Analysis of weighted MI data found that, following all stages of adjustment, compared to the White British group, Black African (OR 0.67, CI 0.63–0.71), Asian (OR 0.65, CI 0.61–0.69) and Mixed ethnic groups (OR 0.80, CI 0.76–0.84) were less likely to self-refer than be referred through their GP (see Table 2). This was also found in the complete case data.

**Table 2.** Association between ethnic groups and method of referral to IAPT services treatment [referral by general practitioner (GP) is the reference]

	GP		Self-referral								Secondary care								Community services							
					Unadjusted		Adjusted for age, gender and year of referral		Adjusted for age, gender, year of referral and borough				Unadjusted		Adjusted for age, gender and year of referral		Adjusted for age, gender, year of referral and borough				Unadjusted		Adjusted for age, gender and year of referral		Adjusted for age, gender, year of referral and borough	
Complete Case	*N*	%	*N*	%	RRR	CI	RRR	CI	RRR	CI	*N*	%	RRR	CI	RRR	CI	RRR	CI	*N*	%	RRR	CI	RRR	CI	RRR	CI
Ethnicity																										
White British	18 043	49.5	16 770	46.0	1.00	1.00–1.00	1.00	1.00–1.00	1.00	1.00–1.00	1044	2.9	1.00	1.00–1.00	1.00	1.00–1.00	1.00	1.00–1.00	611	1.7	1.00	1.00–1.00	1.00	1.00–1.00	1.00	1.00–1.00
Black Caribbean	4068	48.9	3693	44.4	0.98	0.93–1.03	0.98	0.93–1.03	0.87	0.82–0.92	278	3.3	1.18	1.03–1.35	1.23	1.08–1.42	1.14	1.00–1.31	287	3.4	2.08	1.80–2.41	2.36	2.04–2.73	1.92	1.65–2.23
Black African	1912	55.0	1418	40.8	0.80	0.74–0.86	0.77	0.72–0.83	0.92	0.85–1.00	85	2.4	0.77	0.61–0.96	0.85	0.68–1.06	1.02	0.81–1.28	62	1.8	0.96	0.73–1.25	1.04	0.79–1.35	1.37	1.04–1.80
Black Other	798	50.2	668	42.0	0.90	0.81–1.00	1.03	0.93–1.15	0.98	0.87–1.11	52	3.3	1.13	0.84–1.50	1.29	0.97–1.73	1.16	0.87–1.56	72	4.5	2.66	2.07–3.44	3.30	2.55–4.28	3.06	2.33–4.01
Asian	2641	51.6	2182	42.6	0.89	0.84–0.94	0.84	0.79–0.90	0.82	0.77–0.88	182	3.6	1.19	1.01–1.40	1.27	1.07–1.49	1.19	1.01–1.41	118	2.3	1.32	1.08–1.61	1.31	1.07–1.60	1.35	1.10–1.66
Mixed	4551	52.8	3745	43.4	0.89	0.84–0.93	0.90	0.86–0.95	0.89	0.84–0.94	218	2.5	0.83	0.71–0.96	0.90	0.77–1.04	0.91	0.78–1.06	113	1.3	0.73	0.60–0.90	0.81	0.66–0.99	0.77	0.63–0.95
White Other	2139	49.0	1993	45.7	1.00	0.94–1.07	0.96	0.90–1.03	0.93	0.87–1.01	100	2.3	0.81	0.65–1.00	0.97	0.78–1.20	0.94	0.76–1.17	133	3.0	1.84	1.51–2.23	1.87	1.53–2.27	1.81	1.48–2.21
Other	764	49.6	694	45.1	0.98	0.88–1.09	1.02	0.92–1.14	1.01	0.90–1.14	41	2.7	0.93	0.67–1.28	1.00	0.73–1.39	0.98	0.71–1.35	41	2.7	1.58	1.15–2.19	1.66	1.19–2.31	1.64	1.16–2.30
*Weighted MI*																										
Ethnicity																										
White British	–	49.5	–	46.0	1.00	1.00–1.00	1.00	1.00–1.00	1.00	1.00–1.00	–	2.9	1.00	1.00–1.00	1.00	1.00–1.00	1.00	1.00–1.00	–	1.7	1.00	1.00–1.00	1.00	1.00–1.00	1.00	1.00–1.00
Black Caribbean	–	48.9	–	44.4	0.98	0.93–1.03	0.98	0.93–1.03	0.86	0.82–0.91	–	3.3	1.18	1.04–1.35	1.24	1.08–1.42	1.16	1.01–1.33	–	3.4	2.08	1.80–2.41	2.39	2.06–2.76	1.92	1.65–2.24
Black African	–	66.2	–	28.2	0.46	0.43–0.49	0.45	0.42–0.48	0.67	0.63–0.71	–	3.9	1.01	0.87–1.16	1.02	0.88–1.18	1.25	1.07–1.45	–	1.8	0.79	0.64–0.98	0.81	0.66–1.01	1.77	1.43–2.19
Black Other	–	62.3	–	30.3	0.52	0.48–0.58	0.55	0.50–0.60	0.68	0.62–0.76	–	3.9	1.09	0.87–1.35	1.14	0.92–1.42	1.21	0.97–1.50	–	3.4	1.62	1.26–2.08	1.86	1.46–2.37	2.62	2.03–3.38
Asian	–	61.0	–	32.2	0.57	0.54–0.60	0.54	0.51–0.58	0.65	0.61–0.69	–	4.3	1.23	1.08–1.40	1.25	1.10–1.42	1.24	1.08–1.41	–	2.4	1.15	0.97–1.37	1.12	0.94–1.33	1.64	1.38–1.94
Mixed	–	56.2	–	39.3	0.75	0.72–0.79	0.76	0.72–0.80	0.80	0.76–0.84	–	2.9	0.89	0.78–1.03	0.95	0.82–1.10	1.00	0.87–1.16	–	1.6	0.83	0.69–1.00	0.91	0.75–1.09	0.98	0.81–1.19
White Other	–	54.4	–	39.9	0.79	0.74–0.84	0.75	0.70–0.81	0.81	0.75–0.87	–	2.8	0.89	0.72–1.10	1.06	0.85–1.31	1.07	0.86–1.33	–	2.9	1.59	1.32–1.91	1.61	1.34–1.94	1.85	1.52–2.24
Other	–	55.9	–	38.1	0.73	0.66–0.82	0.75	0.67–0.84	0.83	0.74–0.94	–	3.4	1.05	0.79–1.42	1.08	0.81–1.45	1.08	0.80–1.45	–	2.6	1.35	0.96–1.90	1.39	0.99–1.95	1.67	1.17–2.38

Weighted MI is the process of replacing missing data with substituted values as informed by complete data and marginal population level data. It is used here to address missing ethnicity data.

Numbers (*n*), percentages (%),RRRs and 95% CI are shown.

Though not detected in the complete case or MICE imputed datasets, the weighted MI data also indicated that the Black Other (OR 0.68, CI 0.62–0.76), White Other (OR 0.81, CI 0.75–0.87) and Other (OR 0.83, CI 0.74–0.94) ethnic groups were also less likely to self-refer than be referred through their GP compared to the White British ethnic group.

#### Secondary care

Analysis of weighted MI data found that, compared to the White British group, Asian (OR 1.24, CI 1.08–1.41) and Black Caribbean (OR 1.16, CI 1.01–1.33) groups were more likely to be referred to IAPT via secondary care than their GP following all levels of adjustment. This was found in both complete case and weighted MI datasets.

#### Community services

Analysis of the weighted MI dataset found that, compared to the White British service users, Black Caribbean (OR 1.92, CI 1.65–2.24), Black Other (OR 2.62, CI 2.03–3.38) and White Other (OR 1.85, CI 1.52–2.24) groups were more likely to be referred through community services than via their GP following all levels of adjustment. Black African (OR 1.77, CI 1.43–2.19) and Asian groups (OR 1.64, CI 1.38–1.94) were also more likely to be referred through community services in fully adjusted models. This was also found in the complete case data.

Though not detected in the weighted MI data, both complete case data and MICE imputed data found that the Mixed ethnic group was less likely to be referred through community services (OR 0.77, CI 0.63–0.95).

Due to low cell count, interaction effects to identify whether borough moderates the association between ethnicity and referral source could not be tested for.

#### Variations in assessment receipt

Compared to the White British ethnic group, analysis of both the complete case and the weighted MI datasets indicated that the Black Caribbean, Black African, Black Other, Asian, Mixed and White Other ethnic groups were less likely to receive an assessment following referral (see Table 3). These associations remained significant following all levels of adjustment.

**Table 3.** Associations between ethnic groups and receiving an assessment after being referred to IAPT with the use of logistic regression analysis

	Assessment received		Unadjusted		Adjusted for age, gender and year of referral		Adjusted for age, gender, year of referral and borough	
Complete cases	*n*	%	OR	CI	OR	CI	OR	CI
Ethnicity								
White British	27 607	75.4	1.00	1.00–1.00	1.00	1.00–1.00	1.00	1.00–1.00
Black Caribbean	5948	71.1	0.80	0.76–0.85	0.79	0.74–0.83	0.81	0.77–0.86
Black African	2427	69.3	0.74	0.68–0.80	0.72	0.67–0.78	0.75	0.69–0.81
Black Other	1054	65.9	0.63	0.57–0.70	0.72	0.64–0.80	0.68	0.61–0.76
Asian	3776	73.3	0.90	0.84–0.96	0.87	0.81–0.93	0.87	0.81–0.93
Mixed	6265	72.4	0.85	0.81–0.90	0.87	0.83–0.92	0.89	0.84–0.94
White Other	3080	70.3	0.77	0.72–0.83	0.82	0.76–0.88	0.83	0.77–0.89
Other	1185	76.6	1.07	0.95–1.20	1.10	0.97–1.24	1.10	0.97–1.25
*Weighted MI*								
Ethnicity								
White British	–	75.3	1.00	1.00–1.00	1.00	1.00–1.00	1.00	1.00–1.00
Black Caribbean	–	71.0	0.80	0.76–0.84	0.78	0.74–0.82	0.78	0.73–0.82
Black African	–	43.4	0.25	0.24–0.27	0.24	0.22–0.25	0.24	0.22–0.25
Black Other	–	45.0	0.27	0.25–0.29	0.27	0.25–0.29	0.26	0.23–0.28
Asian	–	51.4	0.35	0.33–0.36	0.33	0.31–0.35	0.34	0.32–0.36
Mixed	–	64.0	0.58	0.55–0.61	0.58	0.55–0.61	0.56	0.53–0.59
White Other	–	59.4	0.48	0.45–0.51	0.50	0.47–0.53	0.50	0.47–0.53
Other	–	62.5	0.55	0.49–0.61	0.54	0.48–0.60	0.55	0.49–0.61

Weighted MI is the process of replacing missing data with substituted values as informed by complete data and marginal population level data. It is used here to address missing ethnicity data.

Numbers (*n*), percentages (%),OR and 95% CI are shown.

Analysis of the weighted MI dataset also indicated that the Other ethnic group was significantly less likely to receive an assessment following all levels of adjustment (OR 0.55, CI 0.49–0.61). This is in contrast to the analysis of the complete case dataset which found a positive non-significant association (OR 1.10, CI 0.97–1.25) – these findings from the complete case analysis are supported by the findings from the MICE imputed dataset (see online Supplementary Material D).

Borough of service was found to significantly moderate the effect of ethnicity on receiving an assessment (*p* < 0.01, χ^2^ = 71, df = 21).

#### Variations in treatment receipt

Findings from both the complete case and weighted MI datasets indicate that, among service users who received an assessment, compared to the White British group all other ethnic groups (with the exception of the Mixed ethnic group) were less likely to receive treatment (see Table 4). The Mixed ethnic group was only significantly less likely to receive treatment after adjusting for age, gender, year of referral and borough. For other levels of adjustment this association was non-significant for the Mixed ethnic group in both complete case and weighted MI datasets (OR 0.93, CI 0.88–1.00).

**Table 4.** Associations between ethnic group and treatment receipt among those assessed with the use of logistic regression analysis

	Treatment received (among those assessed)		Unadjusted		Adjusted for age, gender and year of referral		Adjusted for age, gender, year of referral and borough	
Complete cases	*n*	%	OR	CI	OR	CI	OR	CI
Ethnicity								
White British	19 995	72.4	1.00	1.00–1.00	1.00	1.00–1.00	1.00	1.00–1.00
Black Caribbean	3975	66.8	0.77	0.72–0.81	0.76	0.72–0.81	0.72	0.68–0.77
Black African	1605	66.1	0.74	0.68–0.81	0.73	0.67–0.80	0.67	0.61–0.73
Black Other	683	64.8	0.70	0.62–0.80	0.63	0.55–0.72	0.65	0.57–0.75
Asian	2514	66.6	0.76	0.71–0.82	0.78	0.72–0.84	0.81	0.75–0.87
Mixed	4582	73.1	1.04	0.97–1.10	1.00	0.94–1.07	0.93	0.88–1.00
White Other	2078	67.5	0.79	0.73–0.86	0.81	0.75–0.88	0.80	0.74–0.87
Other	800	67.5	0.79	0.70–0.90	0.76	0.67–0.86	0.77	0.68–0.88
Weighted MI								
Ethnicity								
White British	–	72.4	1.00	1.00–1.00	1.00	1.00–1.00	1.00	1.00–1.00
Black Caribbean	–	66.8	0.77	0.72–0.81	0.76	0.72–0.81	0.72	0.68–0.77
Black African	–	67.7	0.80	0.74–0.86	0.78	0.72–0.84	0.77	0.71–0.84
Black Other	–	65.7	0.73	0.65–0.82	0.67	0.59–0.75	0.73	0.64–0.83
Asian	–	66.4	0.75	0.70–0.81	0.77	0.71–0.82	0.83	0.77–0.89
Mixed	–	72.8	1.02	0.96–1.09	0.98	0.92–1.05	0.93	0.88–0.99
White Other	–	67.4	0.79	0.73–0.85	0.81	0.75–0.88	0.82	0.75–0.89
Other	–	67.6	0.79	0.70–0.90	0.76	0.67–0.86	0.79	0.70–0.90

Weighted MI is the process of replacing missing data with substituted values as informed by complete data and marginal population level data. It is used here to address missing ethnicity data.

Numbers (*n*), percentages (%),OR and 95% CI are shown.

Borough was not found to significantly moderate the effect of ethnicity on receiving treatment (*p* > 0.05, χ^2^ = 32, df = 21).

#### Reason for not receiving assessment for treatment

Service users may not receive an assessment or treatment for a variety of reasons; either they did not attend assessment/treatment or dropped out, were discharged, declined treatment, treatment was not suitable for them, or they were referred elsewhere. Percentages across all ethnic groups are not dissimilar (as shown in Table 5). However, in terms of those in the sample who did not receive an assessment, Black African service users had the highest percentage for declining as assessment out of all ethnic groups (26.5%), and Black Other service users had the highest percentage for being referred elsewhere out of all ethnic groups (19%). In terms of those who did not receive treatment following an assessment, no major disparities were shown between ethnic groups.

**Table 5.** Available data on reason for end-of-care pathway

	Did not attend or dropped out		Discharged		Declined		Treatment not suitable		Referred elsewhere^a^		Other	
	*n*	%	*n*	%	*n*	%	*n*	%	*n*	%	*n*	%
End of care pathway for those who did not receive an assessment^b^												
Ethnicity												
White British	4731	52.4	616	6.8	1722	19.1	766	8.5	1061	11.7	137	1.5
Black Caribbean	1226	50.4	149	6.1	506	20.8	172	7.1	346	14.2	34	1.4
Black African	508	47.0	79	7.3	287	26.5	85	7.9	102	9.4	20	1.9
Black Other	299	54.7	47	8.6	70	12.8	23	4.2	104	19.0	4	0.7
Asian	731	53.1	122	8.9	223	16.2	110	8.0	141	10.2	50	3.6
Mixed	1155	48.1	193	8.0	512	21.3	237	9.9	248	10.3	54	2.3
White Other	674	51.6	103	7.9	255	19.5	103	7.9	154	11.8	18	1.4
Other	194	53.3	28	7.7	58	15.9	23	6.3	46	12.6	15	4.1
End of care pathway for those who were assess but did not receive a treatment^c^												
Ethnicity												
White British	2697	35.4	1352	17.8	945	12.4	580	7.6	493	6.5	1545	20.3
Black Caribbean	761	38.6	252	12.8	193	9.8	131	6.6	142	7.2	494	25.0
Black African	279	33.9	127	15.5	87	10.6	72	8.8	63	7.7	194	23.6
Black Other	136	36.7	70	18.9	32	8.6	30	8.1	26	7.0	77	20.8
Asian	373	29.6	219	17.4	172	13.6	93	7.4	60	4.8	345	27.3
Mixed	635	37.7	297	17.6	201	11.9	110	6.5	127	7.5	313	18.6
White Other	370	36.9	153	15.3	99	9.9	67	6.7	78	7.8	235	23.5
Other	132	34.3	56	14.5	37	9.6	34	8.8	30	7.8	96	24.9

a e.g. specialist service or community mental health team.

b *n* = 18 541 due to missing ethnicity data.

c *n* = 15 110 due to missing ethnicity data.

## Discussion

This study utilised electronic IAPT records to identify ethnic inequalities in the method of referral to IAPT services and whether the odds of receiving an assessment and/or initiating treatment varied by ethnicity. The analysis in this study was restricted to the four south London boroughs that fall within the remit of an NHS foundation trust that specialises in and is the sole provider for mental health services in these areas. These boroughs are ethnically diverse and have a greater number of Black Caribbean residents than other London boroughs. This study was able to further examine where disparities in access to and uptake of mental health care for CMDs are experienced by racial and ethnic minority service users, and importantly, highlighted differences between these groups through disaggregating ethnicity. Overall, our findings indicate that racial and ethnic minority groups were less likely to self-refer to IAPT than the White British group and were more likely to be referred via community services. Most racial and ethnic minority groups were also less likely to receive an assessment after being referred and those assessed were also less likely to receive a treatment session than the White British group.

### Method of referral

In contrast to literature demonstrating that self-referral may improve access to IAPT for racial and ethnic minority groups (Brown et al., 2014; Parry et al., 2011), we found many racial and ethnic minority groups to be less likely to self-refer than the White British group; contradicting our first hypothesis.

Disparities in referral pathways may be attributable to a mix of structural and cultural barriers. Self-referral to IAPT is commonly advised and sometimes expected by primary care clinicians as it allows their service users to ‘take ownership’ of their recovery (Thomas et al., 2020). However, qualitative interviews with low-income primary care service users highlighted that being advised to self-refer this could make them feel dismissed or invalidated by their GP after building the courage to seek help for their mental health (Thomas et al., 2020). Further, completing a self-referral via telephone call or online form could seem a challenging task to those dealing with difficulties such as low mood and anxiety. These experiences could exacerbate feelings of disconnect between GP and service user and may lead to individuals not self-referring as advised. Such experiences may also have increased detrimental impact for racial and ethnic minority service users. Literature shows these groups are already less likely to seek help for CMDs from primary care than White ethnic groups (Cooper et al., 2013), may mistrust mental health services and professionals as a result of discrimination from the healthcare system, or may have previously experienced culturally insensitive or naïve interactions with health professionals (Bhui, Warfa, Edonya, McKenzie, & Bhugra, 2007; Henderson et al., 2015; Memon et al., 2016).

Additionally, cultural beliefs about mental health among some racial and ethnic groups can act as a barrier to care. For example, Black African women with experiences of depression were found to have thought the disorder was less serious and less amenable to psychological treatment than White British women (Brown, Boardman, Whittinger, & Ashworth, 2010). Some racial and ethnic minority groups, for example South Asian, may be less likely to perceive mental health problems as medical disorders that can be treated professionally, instead sometimes being attributable to the will of God or poor parenting (Rethink, 2010). We found racial and ethnic minority service users were more likely to have been referred to IAPT via community services, such as a government funded employment agency, voluntary organisations, education providers or criminal justice, than White British service users; this was especially pertinent for Black Caribbean and Black Other ethnic groups. This may be reflective of structural racism generating greater feelings of mistrust towards mental health services, with previous literature showing Black and African Caribbean groups to be over-represented in mental health services, experience worse outcomes and to be over four-times more likely to be detained under the Mental Health Act than White individuals (Bhui et al., 2003; McKenzie, 2007; NHS Digital, 2018b; Sharpley, Hutchinson, McKenzie, & Murray, 2001). However, it may also reflect efforts by IAPT services to liaise with community services to address the under-referral of racial or ethnic minority individuals and highlights the successful work of community services at supporting access to treatment as well as the important role they can play in ensure the health needs of all populations are met.

### Assessment and treatment

In fully adjusted models in both weighted MI and complete case datasets, almost all racial and ethnic minority groups had decreased odds of both receiving an assessment and of receiving at least one treatment session following assessment compared with those in the White British group. This supports our second and third hypotheses that racial and ethnic minority groups would be less likely than the White British group to receive both an assessment and a treatment session, and also supports previous literature that has found racial and ethnic minority groups to be less likely to receive any type of psychological treatment, medication or counselling (Cooper et al., 2013; Mcmanus et al., 2016; Sizmur & McCulloch, 2016), or to be referred to specialist mental health services (Bhui et al., 2003). Similar proportions of service users across all racial and ethnic minority groups did not attend or dropped out of offered treatment, declined treatment or were referred elsewhere, giving no indication of disparities by ethnicity in reasons for not receiving treatment. Previous literature suggests that stigma around mental illness in certain cultures may result in treatment avoidance due to shame, fear or secrecy (Alvidrez, Snowden, & Kaiser, 2008; Rethink, 2010; Shefer et al., 2013). However, it is important to note that discriminatory processes, structures and attitudes exist within mental health care that impact care quality and appropriateness for racial and ethnic minority service users (Joint Commissioning Panel for Mental Health, 2014). Limited research exists evidencing a positive relationship between cultural competency training and improved experiences for racial and ethnic minority health service users (Bennett & Keating, 2009; Healey et al., 2017; Lie, Lee-Rey, Gomez, Bereknyei, & Braddock, 2011). Further, training in cultural competency may allude that inequality is due to the individual's cultural difference and not structural racial bias. IAPT must work towards addressing structural barriers to care, emphasise active anti-racist professional practice and allow for the exploration of racial inequality within their service (Bennett & Keating, 2009; Cénat, 2020). Addressing barriers to treatment are important; treatment avoidance or delay can lead to worsened CMD symptoms so that the level of severity becomes too high for the scope of IAPT practice.

The moderating effect of borough on the association between ethnicity and assessment suggests potentially unequal provision of care for different ethnic groups across the four boroughs that comprise SLaM. This would imply that both the service user's racial or ethnic background and their area of residence impacts the odds of entering the service. This interaction between ethnicity and borough must be addressed through substantial structural changes implemented across SLaM. Addressing the high proportion of missing ethnicity data from IAPT services in Croydon (30%) and Southwark (34%) – considerably higher than Lewisham (6%) and Lambeth (8%) – would be the first step in addressing the problem of unequal provision.

### Strengths and limitations

Using a large dataset, this study demonstrated variation in the way that IAPT service users enter services by ethnicity, and that racial and ethnic minority service users are less likely to receive psychological treatment in IAPT services in four south London boroughs. However, explanations for our results remain speculative and it is unknown whether these individuals received psychological treatment elsewhere or not at all. Unfortunately, socio-economic data (to contextualise our analysis) and mental health prior to treatment (to establish need) were either unavailable to us through IAPT or the large amount of missing data. There is no information available to us in IAPTus about existing mental health diagnoses for those who are referred, we also have no information on those with a mental health need who were not referred to IAPT. Therefore, it is unknown whether these factors may have influenced individual's ability to engage with IAPT services.

We used electronic health record data to identify our dataset, which meant our study was dependent upon IAPT clinicians' input of accurate data. Missing data were an issue with this study, with 18.5% of service users not having data recorded for their ethnicity. This is unfortunately a common issue when utilising healthcare records, despite ethnicity being an incredibly important factor when examining healthcare provision and inequalities. To address this issue, ethnicity data were imputed using two different techniques and reported alongside the complete dataset. Overall results from both these datasets for all research questions were highly similar, increasing the validity of our conclusions.

### Implications

Our findings pose implications for primary care clinicians in facilitating more racial and ethnic minority IAPT referrals, and for IAPT services to consider barriers specific to their racial and ethnic minority service users when engaging with the service. Racial and ethnic minority service users are more likely to be engaging with IAPT services after being referred through more adverse pathways, potentially indicating that their mental health may have been untreated for some time. Further research is needed to examine variation in the number of IAPT referrals by ethnicity and to understand why racial and ethnic minority service users are declining or dropping out of assessments and treatments in IAPT. Clinicians also need to be made aware of this issue and procedures introduced to improve engagement and retention.

Many racial and ethnic minority service users are less likely to self-refer than White British service users, which may mean that they are either unaware of this method of referral, experience more barriers to the use of this method, do not recognise their problems as being appropriate for psychological treatment, or do not trust IAPT services specifically or health services more generally. More effort should be made to gain the trust of racial and ethnic minority service users.

The missing data for ethnicity highlighted in this study is concerning, and more should be done by IAPT to ensure this information is recorded. Analysis of our weighted MI data sometimes highlighted starker ethnic inequalities than that found in the complete case and MICE datasets. This indicates that if the ethnic breakdown of SLaM IAPT service users does reflect that of the population across the boroughs, then due to the amount of missing ethnicity data in IAPT records, the extent of the ethnic inequalities in these boroughs is being obscured.

In addition, the intersections of ethnicity and migration status could not be considered in this study due to country of origin not being recorded in IAPTus. The distinct, and intersecting experiences of migrant service users also from a racial or ethnic minority group may differ greatly from those of British-born, racial and ethnic minority service users, considering migrants face specific barriers to engaging with health services (Gazard, Frissa, Nellums, Hotopf, & Hatch, 2015), potential language limitations (Fountain & Hicks, 2010; Memon et al., 2016) and additional burdens of discrimination or underemployment following migration increasing vulnerability to CMDs (Das-Munshi, Leavey, Stansfeld, & Prince, 2012; Hatch et al., 2016). Changes should be made to IAPTus to capture this information so that IAPT can cater for any specific needs of its migrant service users.

More could be done to ensure mental health services and psychometric measures are adapted to a culturally diverse population. For example, migrant groups may be more likely to require assistance with English language. Potential bias can arise when psychometric scales created from western understandings of mental health are directly translated into other languages (Searight & Searight, 2009). Moreover, whilst SLaM IAPT services do offer interpreters to those who require them, being unable to communicate directly with their therapist can lead to issues detrimental to the therapeutic relationship. This might include problems expressing empathy to the client and impairments in the development of a shared understanding, which may deter from service engagement and lead to a poorer client satisfaction (Bowl, 2007; Fountain & Hicks, 2010; Memon et al., 2016; Tutani, Eldred, & Sykes, 2018). Interpreter availability can also cause delays in assessment and treatment appointments which could have negative effects on access or treatment benefit. As IAPT is a talking therapies service, these factors may influence migrant individuals' ability and desire to attend an assessment or treatment session. For consideration of these and other issues, IAPT have produced a positive practice guide for working with Black, Asian and minority ethnic service users (Beck, Naz, Brooks & Jankowska, 2019).

## Conclusion

This study identifies inequalities in referral source, receipt of an assessment and receipt of treatment for racial and ethnic minority service users. These disparities may be due to a range of cultural, structural and practical barriers along the pathway. Future research making use of qualitative methods would enable exploration of IAPT pathways among racial and ethnic minority service users in more detail, allowing for the identification and exploration of factors and potential mechanisms that are contributing to the generation and perpetuation of these inequalities.

## Supplementary material

For supplementary material accompanying this paper visit https://doi.org/10.1017/S0033291721002518. click here to view supplementary material
