# Supplementary material for: Variations by ethnicity in referral and treatment pathways for IAPT service users in South London
Source: Psychol Med. 2021 Aug 2;53(3):1084–95. doi: 10.1017/S0033291721002518 (PMC9976018; doi:10.1017/S0033291721002518)
Supplement: Supplementary file 1 [file S0033291721002518sup001.docx]

**Supplementary Material A**

|  | **White British** | | **Black Caribbean** | | **Black African** | | **Black Other** | | **Asian** | | **White Other** | | **Mixed** | | **Other** | | | **Total** | |
| --- | --- | --- | --- | --- | --- | --- | --- | --- | --- | --- | --- | --- | --- | --- | --- | --- | --- | --- | --- |
|  | **N** | **%** | **N** | **%** | **N** | **%** | **N** | **%** | **N** | **%** | **N** | **%** | **N** | **%** | **N** | **%** | **N** | | **%** |
| **Borough** |  |  |  |  |  |  |  |  |  |  |  |  |  |  |  |  |  | |  |
| *Croydon* | 5940 | 16.2 | 1152 | 13.7 | 503 | 14.3 | 187 | 11.7 | 1236 | 24 | 900 | 10.4 | 710 | 16.2 | 309 | 19.9 | 10937 | | 15.7 |
| *Lambeth* | 12712 | 34.7 | 3337 | 39.8 | 817 | 23.3 | 671 | 41.9 | 1708 | 33.1 | 2997 | 34.6 | 1584 | 36.1 | 552 | 35.6 | 24378 | | 34.9 |
| *Lewisham* | 10179 | 27.8 | 2528 | 30.2 | 1427 | 40.7 | 338 | 21.1 | 1206 | 23.4 | 2967 | 34.2 | 1250 | 28.5 | 374 | 24.1 | 20269 | | 29 |
| *Southwark* | 7809 | 21.3 | 1364 | 16.3 | 761 | 21.7 | 405 | 25.3 | 1003 | 19.5 | 1800 | 20.8 | 843 | 19.2 | 314 | 20.3 | 14299 | | 20.5 |
| **Age** |  |  |  |  |  |  |  |  |  |  |  |  |  |  |  |  |  | |  |
| *16-24* | 6289 | 17.2 | 1573 | 18.8 | 648 | 18.5 | 315 | 19.7 | 805 | 15.6 | 1044 | 12 | 1305 | 29.7 | 221 | 14.3 | 12200 | | 17.5 |
| *25-34* | 12837 | 35 | 2309 | 27.6 | 1009 | 28.8 | 470 | 29.4 | 1842 | 35.7 | 3358 | 38.8 | 1602 | 36.5 | 483 | 31.2 | 23910 | | 34.2 |
| *35-44* | 7047 | 19.2 | 1697 | 20.2 | 887 | 25.3 | 335 | 20.9 | 1300 | 25.2 | 2334 | 26.9 | 805 | 18.3 | 397 | 25.6 | 14802 | | 21.2 |
| *45-54* | 5580 | 15.2 | 1931 | 23 | 667 | 19 | 330 | 20.6 | 684 | 13.3 | 1146 | 13.2 | 470 | 10.7 | 298 | 19.2 | 11106 | | 15.9 |
| *55-64* | 3003 | 8.2 | 651 | 7.8 | 232 | 6.6 | 112 | 7 | 347 | 6.7 | 513 | 5.9 | 164 | 3.7 | 97 | 6.3 | 5119 | | 7.3 |
| *65+* | 1884 | 5.1 | 220 | 2.6 | 65 | 1.9 | 39 | 2.4 | 175 | 3.4 | 269 | 3.1 | 41 | 0.9 | 53 | 3.4 | 2746 | | 3.9 |
| **Gender** |  |  |  |  |  |  |  |  |  |  |  |  |  |  |  |  |  | |  |
| *Male* | 14395 | 39.3 | 2589 | 30.9 | 1178 | 33.6 | 471 | 29.5 | 1954 | 38 | 2811 | 32.5 | 1413 | 32.3 | 561 | 36.3 | 25372 | | 36.3 |
| *Female* | 22217 | 60.7 | 5787 | 69.1 | 2325 | 66.4 | 1128 | 70.5 | 3194 | 62 | 5844 | 67.5 | 2967 | 67.7 | 986 | 63.7 | 44448 | | 63.7 |
| **PHQ9 caseness** |  |  |  |  |  |  |  |  |  |  |  |  |  |  |  |  |  | |  |
| *Non-caseness* | 7700 | 25.1 | 1172 | 17.2 | 473 | 16.1 | 196 | 16.4 | 824 | 19.8 | 1590 | 22.1 | 629 | 17.8 | 216 | 16.7 | 12800 | | 22.1 |
| *Caseness* | 23016 | 74.9 | 5630 | 82.8 | 2473 | 83.9 | 996 | 83.6 | 3342 | 80.2 | 5603 | 77.9 | 2906 | 82.2 | 1079 | 83.3 | 45045 | | 77.9 |
| **GAD-7 caseness** |  |  |  |  |  |  |  |  |  |  |  |  |  |  |  |  |  | |  |
| *Non-caseness* | 5666 | 18.5 | 1124 | 16.5 | 456 | 15.5 | 202 | 17 | 676 | 16.2 | 1171 | 16.3 | 528 | 15 | 176 | 13.6 | 9999 | | 17.3 |
| *Caseness* | 25040 | 81.5 | 5672 | 83.5 | 2487 | 84.5 | 989 | 83 | 3486 | 83.8 | 6016 | 83.7 | 3002 | 85 | 1120 | 86.4 | 47812 | | 82.7 |
| **Referral source*** |  |  |  |  |  |  |  |  |  |  |  |  |  |  |  |  |  | |  |
| *Primary care* | 18043 | 49.5 | 4068 | 48.9 | 1912 | 55 | 798 | 50.2 | 2641 | 51.6 | 4551 | 52.8 | 2139 | 49 | 764 | 49.6 | 34916 | | 50.2 |
| *Self-referral* | 16770 | 46 | 3693 | 44.4 | 1418 | 40.8 | 668 | 42 | 2182 | 42.6 | 3745 | 43.4 | 1993 | 45.7 | 694 | 45.1 | 31163 | | 44.8 |
| *Secondary care* | 1044 | 2.9 | 278 | 3.3 | 85 | 2.4 | 52 | 3.3 | 182 | 3.6 | 218 | 2.5 | 100 | 2.3 | 41 | 2.7 | 2000 | | 2.9 |
| *Community services* | 611 | 1.7 | 287 | 3.4 | 62 | 1.8 | 72 | 4.5 | 118 | 2.3 | 113 | 1.3 | 133 | 3 | 41 | 2.7 | 1437 | | 2.1 |
| **Assessment received** |  |  |  |  |  |  |  |  |  |  |  |  |  |  |  |  |  | |  |
| *No* | 9033 | 24.7 | 2433 | 29 | 1081 | 30.8 | 547 | 34.2 | 1377 | 26.7 | 2399 | 27.7 | 1307 | 29.8 | 364 | 23.5 | 18541 | | 26.5 |
| *Yes* | 27607 | 75.3 | 5948 | 71 | 2427 | 69.2 | 1054 | 65.8 | 3776 | 73.3 | 6265 | 72.3 | 3080 | 70.2 | 1185 | 76.5 | 51342 | | 73.5 |
| **Treatment received** |  |  |  |  |  |  |  |  |  |  |  |  |  |  |  |  |  | |  |
| *No* | 7612 | 27.6 | 1973 | 33.2 | 822 | 33.9 | 371 | 35.2 | 1262 | 33.4 | 1683 | 26.9 | 1002 | 32.5 | 385 | 32.5 | 15110 | | 29.4 |
| *Yes* | 19995 | 72.4 | 3975 | 66.8 | 1605 | 66.1 | 683 | 64.8 | 2514 | 66.6 | 4582 | 73.1 | 2078 | 67.5 | 800 | 67.5 | 36232 | | 70.6 |
| *Community services include voluntary sector organisations, government service providers, education providers and criminal justice referrals (prison and probation services)* | | | | | | | | | | | | | | | | | | | |
| **Missing referral method data on n=462* | | | | | | | | | | | | | | | | | | | |

## Supplementary Material B

As a minimum, IAPT services provide treatment for people with common mental health problems, including; depression, generalised anxiety disorder, social anxiety disorder, panic disorder, agoraphobia, OCD, phobias, PTSD, health anxiety, and body dysmorphic disorder. The following criteria was in use as of early 2021.

**Southwark Talking Therapies exclusion criteria:**

Generally we do not see people whose needs will be better met elsewhere by a specialist service and/or their other difficulties would be an obstacle to effective treatment in our service, e.g.

- Level of risk that is not manageable within this service
- Addiction problem that is the presenting problem or would be an obstacle to effective treatment in our service
- Eating disorder (unless relatively mild binge eating)
- Current symptoms of psychosis or significant vulnerability to relapse due to history of psychosis (unless diagnosis doubtful or a long time ago with considerable period of stability since)
- Bipolar affective disorder (same caveats as psychosis)
- Borderline or antisocial personality disorder

Patients should not be receiving psychological therapy from another service or therapist at the same time as being seen in our service, although exceptions may be made occasionally, e.g. family therapy.

<https://slam-iapt.nhs.uk/southwark/welcome-to-southwark-psychological-therapies-service/contact-us/>

**Lambeth Talking Therapies exclusion criteria:**

- Those who have a diagnosis of psychosis and who have not been stable for the last 6 months. Where the condition is stable, we only provide treatment for difficulties not directly related to the psychosis problem. For example we can treat anxiety / stress but not paranoia.
- Those who have a diagnosis of bipolar and who have not been stable for 6months. Where the condition is stable we can only treat anxiety / stress difficulties, not problems directly related to the bipolar condition eg depression.
- Those who are high risk and seeking immediate crisis support (i.e. feel that they cannot keep themselves safe)
- Those who are seeking assessment and specific treatment for Autism Spectrum Disorder or ADHD
- Those who are seeking support from a psychiatrist for a psychiatric assessment or medication management
- People who have no access to public funds
- People will not guaranteed to be seen by a clinical psychologist specifically
- We are unable to see patients at their home or those who require weekly home visits
- We are unable to provide letters for housing and benefits applications
- We are unable to make panel referrals for Eating disorders service and Psychosexual services directly, we suggest GPs do this if there is clear indication to refer directly or that if unsure if they meet service criteria please call us and we can do this in conjunction with you or see if the person meets our service criteria
- Those who are seeking and already accessing support from another mental health service (except the Lambeth Living Well Network HUB).
- We offer short term interventions, typically of 6 sessions. We are unable to see clients with long term problems that require long term treatment.
- Treatment involves a client being able to attend regularly, be active and make changes. We do not provide general support.

**Croydon Talking Therapies inclusion criteria (no exclusion criteria given):**

Our aim is to help improve the mental well-being of people experiencing difficulties such as:

- Low mood
- Feeling down due to unemployment or difficulties at work
- Sleep problems
- Low self-esteem
- Panic attacks
- Sadness that won’t go away
- Excessive worrying or anxiety
- Relationship or family problems related to how you feel
- Low mood or anxiety related to a medical condition such as diabetes, Irritable bowel Syndrome and Fatigue
- Experience of a traumatic event
- Stress brought on by adjusting to life event
- Loss and grief
- Stress
- Obsessive Compulsive Disorder
- Phobias

<https://slam-iapt.nhs.uk/croydon/therapy-options/types-of-difficulties/>

**Lewisham Talking Therapies inclusion criteria (no exclusion criteria given):**

Here at IAPT Lewisham, we will assess your current difficulties and will carefully consider if our service is right for you. If not, we will put you in touch with other services in your area that can give you the support that would best suit you.

We offer a range of short-term psychological therapy options aimed to support you with the following difficulties:

- [Depression](http://www.nhs.uk/Conditions/Depression/Pages/Introduction.aspx)
- [Excessive worrying or anxiety](http://www.nhs.uk/Conditions/Anxiety/Pages/Introduction.aspx)
- [Panic attacks](http://www.nhs.uk/conditions/panic-disorder/pages/introduction.aspx)
- [Sleep problems](http://www.nhs.uk/Conditions/Insomnia/Pages/Introduction.aspx)
- [Stress](http://www.nhs.uk/conditions/stress-anxiety-depression/pages/understanding-stress.aspx)
- [Phobia](http://www.nhs.uk/Conditions/stress-anxiety-depression/Pages/dealing-with-fears.aspx)
- [Low self-esteem](http://www.nhs.uk/Livewell/mentalhealth/Pages/Dealingwithlowself-esteem.aspx)
- [Obsessive compulsive disorder](http://www.nhs.uk/conditions/obsessive-compulsive-disorder/pages/introduction.aspx)
- [Low mood due to a long-term health condition for example diabetes or fatigue](http://www.nhs.uk/planners/yourhealth/Pages/Healthcare.aspx)
- [Experience of a traumatic event](http://www.nhs.uk/conditions/post-traumatic-stress-disorder/pages/introduction.aspx)
- [Loss and grief](http://www.nhs.uk/Livewell/emotionalhealth/Pages/Dealingwithloss.aspx)
- [Feeling down due to unemployment or difficulties at work](http://www.nhs.uk/conditions/stress-anxiety-depression/pages/workplace-stress.aspx)
- [Feeling down due to relationship problems or life changes](http://www.nhs.uk/Conditions/stress-anxiety-depression/Pages/low-mood-and-depression.aspx)

## <https://slam-iapt.nhs.uk/lewisham/what-we-offer/>

## Supplementary Material C

**Table S1: IAPT and census data by ethnicity across Lewisham, Southwark, Lambeth and Croyden. Imputed ethnicity data also shown.**

|  |  |  |  |  | ***Imputed data**** | | | |
| --- | --- | --- | --- | --- | --- | --- | --- | --- |
|  | **IAPT** | | **Census data** | | **WMI** | | **MICE** | |
| **Ethnicity** | n | % | n | % | n | % | n | % |
| *White British* | 36640 | 52.4% | 518970 | 42.2% | - | 42.7% | - | 52.7% |
| *Black Caribbean* | 8381 | 12.0% | 109034 | 8.9% | - | 9.8% | - | 11.8% |
| *Black African* | 3508 | 5.0% | 143606 | 11.7% | - | 10.9% | - | 5.0% |
| *Black Other* | 1601 | 2.3% | 51611 | 4.2% | - | 4.1% | - | 2.3% |
| *Asian* | 5153 | 7.4% | 133291 | 10.8% | - | 10.9% | - | 7.5% |
| *Mixed* | 8664 | 12.4% | 85305 | 6.9% | - | 12.4% | - | 12.2% |
| *White Other* | 4387 | 6.3% | 158285 | 12.9% | - | 6.6% | - | 6.2% |
| *Other* | 1549 | 2.2% | 30530 | 2.5% | - | 2.5% | - | 2.2% |
| **Borough, gender and age used as auxiliary variables* | | | | | | | | |

## Supplementary Material D

The analysis reported in the section has been conducted on the IAPT data described in the methods section of the main paper. Missing ethnicity data has been imputed using multiple imputation by chained equations (MICE).

**Table S2: Multinomial regression analysis on MICE data to show association between ethnic groups and method of referral to IAPT services treatment (referral by general practitioner (GP) is the reference). Numbers (n), percentages (%), Relative Risk Ratios (RRR) and 95% confidence intervals (CI) are shown.**

|  | **Self-referral** | | | | | | **Secondary care** | | | | | | **Community services** | | | | | |
| --- | --- | --- | --- | --- | --- | --- | --- | --- | --- | --- | --- | --- | --- | --- | --- | --- | --- | --- |
|  | **Unadjusted** | | **Adjusted for age, gender and year of referral** | | **Adjusted for age, gender, year of referral and borough** | | **Unadjusted** | | **Adjusted for age, gender and year of referral** | | **Adjusted for age, gender, year of referral and borough** | | **Unadjusted** | | **Adjusted for age, gender and year of referral** | | **Adjusted for age, gender, year of referral and borough** | |
|  | ***RRR*** | ***CI*** | ***RRR*** | ***CI*** | ***RRR*** | ***CI*** | ***RRR*** | ***CI*** | ***RRR*** | ***CI*** | ***RRR*** | ***CI*** | ***RRR*** | ***CI*** | ***RRR*** | ***CI*** | ***RRR*** | ***CI*** |
| ***Standard MI*** |  |  |  |  |  |  |  |  |  |  |  |  |  |  |  |  |  |  |
| **Ethnicity** |  |  |  |  |  |  |  |  |  |  |  |  |  |  |  |  |  |  |
| *White British* | 1.00 | 1.00,1.00 | 1.00 | 1.00,1.00 | 1.00 | 1.00,1.00 | 1.00 | 1.00,1.00 | 1.00 | 1.00,1.00 | 1.00 | 1.00,1.00 | 1.00 | 1.00,1.00 | 1.00 | 1.00,1.00 | 1.00 | 1.00,1.00 |
| *Black Caribbean* | 1.02 | 0.97,1.07 | 1.02 | 0.97,1.07 | 0.89 | 0.84,0.94 | 1.14 | 1.00,1.31 | 1.18 | 1.03,1.35 | 1.11 | 0.97,1.27 | 1.90 | 1.65,2.19 | 2.18 | 1.89,2.51 | 1.72 | 1.49,1.99 |
| *Black African* | 0.84 | 0.78,0.90 | 0.81 | 0.76,0.88 | 0.94 | 0.87,1.01 | 0.82 | 0.66,1.03 | 0.89 | 0.71,1.11 | 1.03 | 0.82,1.29 | 0.93 | 0.72,1.22 | 1.01 | 0.77,1.32 | 1.33 | 1.01,1.75 |
| *Black Other* | 0.92 | 0.82,1.02 | 1.01 | 0.91,1.13 | 0.98 | 0.87,1.10 | 1.03 | 0.79,1.34 | 1.16 | 0.89,1.51 | 1.09 | 0.83,1.43 | 2.29 | 1.77,2.96 | 2.81 | 2.17,3.65 | 2.62 | 1.99,3.43 |
| *Asian* | 0.89 | 0.84,0.95 | 0.85 | 0.80,0.91 | 0.86 | 0.80,0.92 | 1.16 | 0.99,1.35 | 1.22 | 1.04,1.42 | 1.14 | 0.97,1.33 | 1.20 | 0.98,1.47 | 1.20 | 0.98,1.46 | 1.27 | 1.03,1.56 |
| *Mixed* | 0.93 | 0.89,0.98 | 0.94 | 0.90,0.99 | 0.91 | 0.86,0.96 | 0.83 | 0.72,0.96 | 0.90 | 0.78,1.04 | 0.92 | 0.80,1.07 | 0.78 | 0.64,0.94 | 0.86 | 0.71,1.04 | 0.80 | 0.66,0.98 |
| *White Other* | 1.01 | 0.95,1.08 | 0.98 | 0.91,1.04 | 0.95 | 0.88,1.02 | 0.81 | 0.66,0.99 | 0.97 | 0.79,1.19 | 0.95 | 0.77,1.17 | 1.65 | 1.37,2.00 | 1.71 | 1.41,2.08 | 1.66 | 1.36,2.02 |
| *Other* | 0.97 | 0.87,1.08 | 1.00 | 0.90,1.11 | 1.00 | 0.88,1.12 | 0.94 | 0.70,1.26 | 1.00 | 0.74,1.35 | 0.96 | 0.71,1.30 | 1.42 | 1.03,1.95 | 1.48 | 1.08,2.05 | 1.47 | 1.04,2.06 |

Table S3: Regression analysis on MICE data to show association between ethnic groups and receiving an assessment after being referred to IAPT. Numbers (n), percentages (%), Odds Ratios (OR) and 95% confidence intervals (CI) are shown.

| ***Standard MI*** |  | **Unadjusted** | | **Adjusted for age, gender and year of referral** | | **Adjusted for age, gender, year of referral and borough** | |
| --- | --- | --- | --- | --- | --- | --- | --- |
|  |  | **OR** | **CI** | **OR** | **CI** | **OR** | **CI** |
| **Ethnicity** |  |  |  |  |  |  |  |
| *White British* | 66.2% | 1.00 | 1.00,1.00 | 1.00 | 1.00,1.00 | 1.00 | 1.00,1.00 |
| *Black Caribbean* | 63.8% | 0.90 | 0.86,0.95 | 0.88 | 0.84,0.93 | 0.86 | 0.82,0.91 |
| *Black African* | 61.7% | 0.82 | 0.77,0.89 | 0.81 | 0.75,0.88 | 0.81 | 0.75,0.88 |
| *Black Other* | 58.7% | 0.73 | 0.65,0.82 | 0.78 | 0.70,0.88 | 0.76 | 0.67,0.85 |
| *Asian* | 63.4% | 0.89 | 0.83,0.94 | 0.87 | 0.81,0.92 | 0.91 | 0.85,0.97 |
| *Mixed* | 64.9% | 0.94 | 0.90,0.99 | 0.95 | 0.90,1.01 | 0.92 | 0.87,0.97 |
| *White Other* | 62.1% | 0.84 | 0.78,0.89 | 0.88 | 0.82,0.94 | 0.88 | 0.83,0.95 |
| *Other* | 66.8% | 1.03 | 0.92,1.15 | 1.04 | 0.93,1.17 | 1.07 | 0.95,1.20 |

Table S4: Regression analysis on MICE data to show association between ethnic groups and being treated (among those who have been assessed). Numbers (n), percentages (%), Odds Ratios (OR) and 95% confidence intervals (CI) are shown.

| ***Standard MI*** |  | **Unadjusted** | | **Adjusted for age, gender and year of referral** | | **Adjusted for age, gender, year of referral and borough** | |
| --- | --- | --- | --- | --- | --- | --- | --- |
|  |  | **OR** | **CI** | **OR** | **CI** | **OR** | **CI** |
| **Ethnicity** |  |  |  |  |  |  |  |
| *White British* | 72.1% | 1.00 | 1.00,1.00 | 1.00 | 1.00,1.00 | 1.00 | 1.00,1.00 |
| *Black Caribbean* | 67.1% | 0.79 | 0.74,0.84 | 0.78 | 0.74,0.83 | 0.74 | 0.70,0.79 |
| *Black African* | 66.4% | 0.76 | 0.70,0.84 | 0.75 | 0.69,0.83 | 0.69 | 0.63,0.76 |
| *Black Other* | 65.0% | 0.72 | 0.63,0.82 | 0.65 | 0.57,0.74 | 0.68 | 0.59,0.77 |
| *Asian* | 66.6% | 0.77 | 0.72,0.83 | 0.79 | 0.74,0.85 | 0.83 | 0.77,0.89 |
| *Mixed* | 72.9% | 1.04 | 0.98,1.11 | 1.01 | 0.95,1.07 | 0.94 | 0.88,1.00 |
| *White Other* | 67.4% | 0.80 | 0.74,0.87 | 0.83 | 0.76,0.90 | 0.82 | 0.75,0.89 |
| *Other* | 67.5% | 0.80 | 0.71,0.91 | 0.77 | 0.68,0.88 | 0.79 | 0.69,0.90 |
